# Effects on clients' daily functioning and common features of reablement interventions: a systematic literature review

**DOI:** 10.1007/s10433-022-00693-3

**Published:** 2022-05-03

**Authors:** Lise E. Buma, Stan Vluggen, Sandra Zwakhalen, Gertrudis I. J. M. Kempen, Silke F. Metzelthin

**Affiliations:** 1 Department of Health Services Research, Care and Public Health Research Institute, Maastricht University, P.O. Box 616, 6200 MD Maastricht, The Netherlands; 2 Living Lab in Ageing and Long-Term Care, Maastricht, The Netherlands; 3 Cicero Zorggroep, P.O. Box 149, 6440 AC Brunssum, The Netherlands

**Keywords:** Reablement, Activities of daily living, Independence, Daily functioning, Person-centred

## Abstract

This systematic review aimed to provide an overview of reablement interventions according to the recently published ReAble definition and their effect on Activities of Daily Living (ADL). In addition, the most common and promising features of these reablement interventions were identified. Four electronic bibliographic databases were searched. Articles were included when published between 2002 and 2020, which described a Randomised or Clinical Controlled Trial of a reablement intervention matching the criteria of the ReAble definition, and had ADL functioning as an outcome. Snowball sampling and expert completion were used to detect additional publications. Two researchers screened and extracted the identified articles and assessed methodological quality; discrepancies were resolved by discussion and arbitration by a third researcher. Twenty relevant studies from eight countries were included. Ten of these studies were effective in improving ADL functioning. Identifying promising features was challenging as an equal amount of effective and non-effective interventions were included, content descriptions were often lacking, and study quality was moderate to low. However, there are indications that the use of more diverse interdisciplinary teams, a standardised assessment and goal-setting method and four or more intervention components (i.e. ADL-training, physical and/or functional exercise, education, management of functional disorders) can improve daily functioning. No conclusions could be drawn concerning the effectiveness on ADL functioning. The common elements identified can provide guidance when developing reablement programmes. Intervention protocols and process evaluations should be published more often using reporting guidelines. Collecting additional data from reablement experts could help to unpack the black box of reablement.

**Supplementary Information:**

The online version contains supplementary material available at 10.1007/s10433-022-00693-3.

## Introduction

With increasing age, older adults often experience functional disability, which is described as difficulty or dependency in the execution of daily functioning or Activities of Daily Living (ADL) (Covinsky et al. 1997; Fried et al. 2004; Lafortune 2007). ADL can be divided into basic self-care skills such as eating, bathing or dressing (bADL); more complex and instrumental activities such as using a telephone, doing the laundry or managing medications (iADL); and advanced culture and gender-specific activities not necessary for independent living such as hobbies, religion and working (aADL) (Reuben and Solomon 1989). Difficulties in executing ADL are associated with poor quality of life, depression, hospitalisation and nursing home placement, and increased disability (Arnau et al. 2016). It is therefore important to optimise older adults' active involvement and participation in daily functioning (Aspinal et al. 2016).

Older adults can generally rely on help from health and social care staff in performing everyday activities. However, these professionals often work in a task-oriented fashion; they are used to doing tasks for or to the individual, rather than doing tasks with them in a more rehabilitative and person-centred manner (Aspinal et al. 2016; Kitson et al. 2014). This task-oriented approach can lead to a downward spiral, with a greater loss of functions and paradoxically greater care consumption (Gingerich 2016; Schuurmans et al. 2020; Whitehead et al. 2015). Therefore, a paradigm shift in health, respectively, social care is needed, which focuses on person-centeredness and promotes older adults' active involvement and participation.

An innovative approach that can guide this shift is reablement. As there was ambiguity on the concept of reablement, a Delphi study was conducted among 81 international experts that saw reablement defined as: “*A person-centred, holistic approach that aims to enhance an individual’s physical and/or other functioning, to increase or maintain their independence in meaningful activities of daily living at their place of residence and to reduce their need for long-term services. Reablement consists of multiple visits and is delivered by a trained and coordinated interdisciplinary team. The approach includes an initial comprehensive assessment followed by regular reassessments and the development of goal-oriented support plans. Reablement supports an individual to achieve their goals, if applicable, through participation in daily activities, home modifications, and assistive devices as well as involvement of their social network. Reablement is an inclusive approach irrespective of age, capacity, diagnosis or setting*” (Metzelthin et al. 2020).

Due to the use of different definitions of reablement before the existence of the ReAble definition, divergent results were found regarding the effectiveness. Reablement has shown positive effects on improving or maintaining ADL and physical functioning, quality of life, and reducing the risk of death or permanent residential care and healthcare costs (Ryburn et al. 2009; Sims-Gould et al. 2017; Tessier et al. 2016; Whitehead et al. 2015), while other reablement reviews have demonstrated no effects, reported a lack of intervention descriptions or could not include studies (Cochrane et al. 2016; Legg et al. 2016; Mjøsund et al. 2020). The contradictory evidence seems to link back to the conceptualisation of reablement. First, existing systematic reviews defined reablement differently, which led to different inclusion criteria and requirements. Consequently, different conclusions were drawn on the effects of reablement. As a result, one systematic review found no indication that reablement led to less dependency in ADL functioning (Cochrane et al. 2016). In contrast, four systematic reviews found that reablement showed positive results in optimising ADL functioning (Resnick et al. 2013; Sims-Gould et al. 2017; Tessier et al. 2016; Whitehead et al. 2015). Second, reablement is often used interchangeably with other interventions; for example, in the review of Sims-Gould et al. (2017), four different types of interventions were included, namely reactivation, restorative, rehabilitation, and reablement. Lastly, another shortcoming is that the studied interventions show great variation in how reablement and its components are applied and shaped in practice. This is highlighted by Doh et al. (2019), who point out the variation of reablement interventions between and even within different countries.

Currently, it is unknown what the evidence on reablement is when this definition is used as a starting point. Given the objective of reablement, increasing independence, it is particularly interesting to look at daily functioning. Therefore, using the ReAble definition as a starting point, this systematic review aims to provide a current overview of reablement interventions internationally, and their effect on clients’ daily functioning, combined with identifying common and possibly promising features. This systematic review is guided by the following three research questions: (1) What are the effects of reablement on daily functioning among individuals in need of care irrespective of age, capacity, diagnosis, or setting?; (2) What are the common features of reablement interventions according to the elements addressed in the ReAble definition (e.g. assessment, goal-setting tools, and staff training)?; and (3) What are the most promising reablement features?

## Methods

A systematic review was conducted following the guidelines published by the Cochrane Collaboration and Preferred Reporting Items for Systematic reviews and META-analyses (PRISMA) statement (Akl 2019; Moher et al. 2009). A review protocol was established a priori and registered with PROSPERO (https://www.crd.york.ac.uk/PROSPERO/, ID CRD42020215245).

### Eligibility criteria

Studies were eligible when the described intervention was in line with the criteria of the ReAble definition (Metzelthin et al. 2020). Therefore, participants included were ≥ 18 years old, and in need of care, irrespective of capacity, diagnosis or setting. Studies were included when interventions aimed to enhance an individual’s physical and/or other types of functioning; increase or maintain independence in meaningful ADLs at the place of residence; or reduce the need for long-term services. The interventions had to be delivered by an interdisciplinary team, include an initial assessment followed by regular assessments and contained a goal-oriented support plan. Interventions were excluded when problem-oriented (e.g. malnutrition, pain, falls); focussed on assessment and/or care management only; not delivered at the place of permanent residence (e.g. group sessions or at a community centre); delivered by different disciplines, but did not include interdisciplinary collaboration and coordination; and when studies compared outpatient with inpatient care. Randomised controlled trials (RCTs) and controlled clinical trials (CCTs) were included if ADL functioning was used as an outcome measure in terms of basic ADL/instrumental ADL/advanced ADL, if reablement was compared to usual care, and when they were published in English or Dutch between 2002 and 2020. The year 2002 was chosen because the study by Tinetti et al. (2002) is the first known study to introduce the term reablement.

### Search strategy

The following electronic bibliographic databases were searched in July 2020 and repeated in July 2021: PubMed, CINAHL (EBSCO), PsycInfo (EBSCO) and the Cochrane Library. An information specialist at Maastricht University verified the search string (see Appendix 1). It used terms relating to or describing the population, intervention, outcome and study design. The search strategy used Medical Subject Headings (MeSH); if MeSH were not available, appropriate keywords were used. The initial search was conducted in PubMed, and search terms were modified if necessary, to make them applicable in other databases. To check the adequacy of the search string, two well-known references (Lewin et al. 2013; Tuntland et al. 2015) were used as key references to check whether they were identified by the initial search. This method is known for optimising the search and assuring that all relevant studies will be identified (Booth 2008; Cooper et al. 2018). *The initial search string did not filter on the ageing population, however, as a result, we found many articles on rehabilitation (that were diagnosis-specific), which did not meet the criteria of the definition. To enhance the specificity of the search results, the choice was made to filter on the ageing population as most studies on reablement have been aimed at this cohort. However, also studies that were not explicitly aimed at older adults were eligible for our systematic review. To guarantee that no relevant studies were missed we conducted snowball sampling by checking the references of the included papers and consulted experts in the field of reablement. The experts were specifically asked for studies that were conducted on younger people.*

### Study selection

All search results from the different databases were merged, after which duplicates were removed. To facilitate the screening of results, the web-based application Rayyan was used (Ouzzani et al. 2016). Two researchers (LB and SM) independently screened the studies on title and abstract. If the inclusion criteria were met, both assessed the full text for eligibility. LB and SM decided independently whether the inclusion criteria were met. Both screened 5% of the studies using the title and abstract first; when the consensus was < 80% overall, an additional 5% was screened, after which at least 80% consensus was reached. Discrepancies were resolved by discussion and, where required, arbitration by a third researcher (SZ). An additional snowball sampling was used on studies included in the final sample (Wohlin 2014). Their reference lists were screened and studies were included according to the screening process described above. Reference lists of existing reviews on reablement (Cochrane et al. 2016; Legg et al. 2016; Resnick et al. 2013; Ryburn et al. 2009; Sims-Gould et al. 2017; Tessier et al. 2016; Whitehead et al. 2015) were checked to ensure that no key publications were missed. After these steps, SZ performed a final check of which studies to include. The authors of the included studies were contacted, as were 39 experts affiliated with the ReAble network (https://reable.auckland.ac.nz/reable-network), with a request to check whether any important studies had been missed. When additional studies were suggested, they were screened for inclusion according to the process described above.

### Data extraction and analyses

All information was extracted using a data extraction template, as shown in Appendix 2, which was created in Microsoft Excel for the current study (Microsoft 2016). Study characteristics (i.e. design, aims, hypotheses and target group), common intervention components (i.e. team composition, duration, assessment, and goal setting) and outcome data concerning the effects on daily functioning were also extracted. Study protocols and additional related publications were also used.

### Risk of bias and quality assessment

The methodological quality of the included studies was assessed by LB and checked by SM using the Critical Appraisal Checklists provided by the Joanna Briggs Institute (JBI) (Tufanaru et al. 2020). The checklist included the following criteria that were assessed: adequate method of randomisation (if applicable), allocation concealment, the similarity of groups at baseline, blinding of participants, personnel and outcome assessors, method of measurement, statistical analyses and appropriate use of trial design. Risk of bias was rated for selection-, performance-, attrition-, detection- and reporting bias. Each aspect of methodological quality, the risk of bias assessment and the overall risk of bias for the entire set of the included studies were reported in tabular and narrative forms for each study. When an answer on the checklist items was "yes", a score of 1 was given, when the answer was "unclear" or "no", a score of 0 was given. The impact of methodological quality of studies was assessed using a narrative synthesis.

## Results

### Study selection

**Fig. 1:**
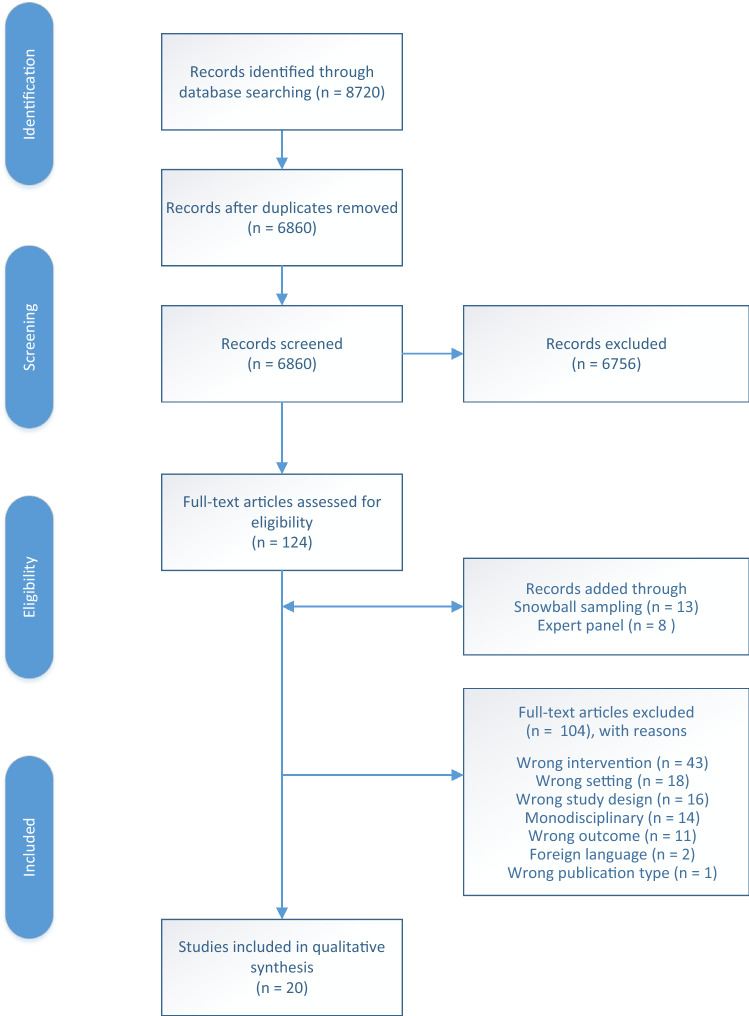
Flow chart study selection process

Searches of the electronic databases were carried out in July 2020 and yielded 7844 articles. A total of 1830 duplicates had been removed. The repeated search in July 2021 yielded an additional 876 unique articles. In total, 6,860 titles and abstracts were screened. Of these titles and abstracts, 104 studies were included for full-text screening. A total of fifteen articles describing fifteen independent intervention studies eventually met the eligibility criteria. An additional snowball sampling of the included studies and systematic reviews on reablement resulted in an extra thirteen studies. Finally, after consulting experts (from the ReAble network and the first authors of included studies), an additional eight studies were obtained. After the full-text screening of the snowball sample and studies suggested by experts, five were included in the final sample (*n* = 20). The flow chart of the screening process according to the PRISMA statement (Moher et al. 2009), including reasons for exclusion, can be found in Fig. 1.

### Study and participant characteristics

The study and participant characteristics of the twenty included studies are shown in Table 1. Sixteen studies were RCTs and four were CCTs. The studies were conducted in eight countries. The twenty studies comprised a total of 6798 participants (range 61–1382), most were female 69.8% (range 21.6–87.5) and had a mean age of 79.5 ± 7.8 years old (range 34.5–87.7). Thirteen studies were conducted in community care and seven studies in institutionalised long-term care. Four studies used the diagnosis of dementia as their main focus group instead of a specific setting.

**Table 1:** Study characteristics of the included studies (*n* = 20)

Author (year)	Study aim	Design and follow-up	Setting	Study population criteria	Sample
United States of America					
Galik et al. (2014)^a^	To determine the effect of Function-Focused Care for the Cognitively Impaired on function, physical activity, behaviour, and falls	Cluster-RCT Follow-up 3 and 6 months	Institutionalised long-term care	• ≥ 55 years old • MMSE-score ≤ 15 • Living in nursing home • Expected to stay in nursing home for at least 6 months	Mean age 83.7 (9.9), 77% female IG *n* = 53 CG *n* = 50
Galik et al. (2015)	To determine the effect of Function-Focused Care for the Cognitively Impaired on function, physical activity, behaviour, and falls	Cluster-RCT Follow-up 3 and 6 months	Institutionalised long-term care (dementia-specific)	• ≥ 55 years old • MMSE-score ≤ 15 • Living in assisted living • Expected to stay in assisted living for at least 6 months	Mean age 83.7 (7.2), 65% female IG *n* = 48 CG *n* = 48
Gitlin et al. (2006)^a^	To determine the effect of a multicomponent intervention to reduce functional difficulties, fear of falling, and home hazards and enhance self-efficacy and adaptive coping in older adults with chronic conditions	RCT Follow-up 6 and 12 months	Community care	• ≥ 70 years old • Cognitively intact • Not receiving home care • Having trouble with 2 iADLs or ≥ 1 ADLs • Not completely dependent or homebound • Not receiving services to address functional problems	Mean age 79 (5.9), 82% female IG *n* = 160 CG *n* = 159
Gitlin et al. (2010)^a^	To determine the effect of a non-pharmacologic, bio behavioural approach (COPE) to support physical function and quality of life for patients with dementia and the well-being of their caregivers	RCT Follow-up 4 and 9 months	Community care	• ≥ 21 years old • Diagnosis of dementia • Needing help with daily activities or having behavioural symptoms • Lived with or within 5 miles of family caregivers • Not having terminal illness, active cancer treatment, more than 3 acute hospitalisations in the past year, diagnosis of schizophrenia, bipolar disorder, dementia secondary to head trauma, MMSE-score of 0 or bed-bound mobility	Mean age 82.4 (8.9), 68% female IG *n* = 102 CG *n* = 107
Resnick et al. (2011)	To determine the effect of Function-Focused Care Assisted Living to alter the decline that AL-clients experience	Cluster-RCT Follow-up 4 and 12 months	Institutionalised long-term care	• ≥ 65 years old • MMSE-score ≥ 11 • Living in assisted living • Not in hospice or rehabilitation	Mean age 87.7 (5.7), 80% female IG *n* = 93 CG *n* = 78
Szanton et al. (2019)^a^	To determine the effect whether a 10-session, home-based, interdisciplinary programme (CAPABLE) reduces disability	RCT Follow-up 5 and 12 months	Community care	• ≥ 65 years old • Cognitively intact • Reporting difficulty with at least 1 ADL or ≥ 2 iADLs • Income < 200% of federal poverty level • Not having active cancer treatment, more than 3 acute hospitalisations in the past year, inability to stand, apartment dwelling, plans to move within a year or use of home-based physical or occupational therapy services at enrolment	Mean age 75.7 (7.6), 88% female IG *n* = 152 CG *n* = 148
Tinetti et al. (2002)^a^	To determine the effect on functional status and the likelihood of remaining at home for persons receiving restorative care	CCT Follow-up every 60 day, and at discharge from homecare (average 35 days)	Community care	• ≥ 65 years old •Receiving home care •At risk for functional decline after acute illness or hospitalisation but potential for maintaining or improving their function •Not having severe cognitive impairment, requiring total assistance with care or bed-bound mobility	Mean age 79.8 (6.9), 59% female IG *n* = 691 CG *n* = 691
New Zealand					
Kerse et al. (2008)	To determine the effect of an activity programme (PIRC) in improving function, quality of life, and falls in older people in residential care	Cluster-RCT Follow-up 6 and 12 months	Institutionalised long-term care	• ≥ 65 years old • Cognitively able to set and remember goals to be achieved • Needing assistance with most iADLs and ≥ 2 ADLs • Can usually ambulate to some degree and feed themselves • Not unable to communicate, having anxiety as main diagnosis, acutely unwell or in case of terminal illness	Mean age 84 (7.0), 75% female IG *n* = 330 CG *n* = 352
King et al. (2012)	To determine the effect of a long-term restorative home care service	Cluster-RCT Follow-up 4 and 7 months	Community care	• ≥ 65 years old • Receiving assistance from the home care agency • Not unable to participate in interviews due to poor health	Mean age 79.4 (6.4), 76% female IG *n* = 93 CG *n* = 93
Parsons et al. (2017)	To determine the effect of a restorative home support service on institutional-free survival in frail older people referred for needs assessment	RCT Follow-up at regular intervals over 24 months, not specified	Community care	• ≥ 65 years old • Assessed at high risk of permanent institutional care • Not needing nursing home placement	Mean age 83.1 (7.5), 61% female IG *n* = 56 CG *n* = 57
Parsons et al. (2020)	To determine the effect whether early Supported Discharge Teams (SDT) for older people admitted to hospital following a fracture enables earlier discharge from hospital and reduces readmissions and healthcare costs	RCT Follow-up 12 months	Hospital to community care	• ≥ 65 years old • Suffered an injury requiring hospital admission • In hospital at time of referral • Not requiring ongoing acute hospital-based treatment	Mean age 80.8 (8.1), 75% female IG *n* = 201 CG *n* = 202
Australia					
Lewin and Vandermeulen (2010)^a^	To determine the effect of Home Independence Programme (HIP) on promoting functional dependency, morale, confidence in performing everyday activities without falling and functional mobility	CCT Follow-up 3 and 12 months	Community care	• ≥ 60 years old • Referred for home care services • Existing home care clients who request an increase in level or amount of service • Not having cognitive impairment, other progressive neurological disorders or when in need for acute or post-acute care	Mean age 79.7 (5.9), 75% female IG *n* = 100 CG *n* = 100
Lewin et al. (2013)^a^	To determine the effect of a new paradigm for home care (HIP)	RCT Follow-up 3 and 12 months	Community care	• ≥ 65 years old • Referred for home care services • Existing home care clients needing an increase of service • Needing assistance with one or more ADLs because of an ongoing disability • Not having terminal illness, cognitive impairment due to dementia or progressive neurological disorder	Mean age 82.3 (7.4), 67% female IG *n* = 375 CG *n* = 375
United Kingdom					
Powell et al. (2002)^a^	To determine the effect of an interdisciplinary community-based outreach rehabilitation after severe traumatic brain injury	RCT Follow-up at end of treatment (average 24 months)	Community care	• Between 16 and 65 years old • Sustained traumatic brain injury • Referred from home or discharge at hospital • With long-term treatment goals to improve independence in activities of daily living, social participation, and psychological wellbeing • Severity of injury was at least moderate • No concurrent neurological disorders	Mean age 34.5 (10.5), 22% female IG *n* = 48 CG *n* = 46
Sackley et al. (2009)	To determine the effect of a programme of physiotherapy and occupational therapy in care home residents with mobility limitations dependent on carers in some ADLs	Cluster-RCT Follow-up 3 and 6 months	Institutionalised long-term care	• Living in a care home • Experiencing limitations in mobility or ADL • BI-score between 6 and 16 • No admissions to hospital, nursing home or hospice	Mean age 85.0 (8.5), 74% female IG *n* = 128 CG *n* = 121
Sweden, Norway, Denmark					
Gronstedt et al. (2013)	To determine the effect of individually tailored physical and daily activities in nursing home residents on ADL and balance	RCT Follow-up 3 months	Institutionalised long-term care	• ≥ 64 years old • In need of daily assistance of minimum one personal ADL due to physical disability • Expected to stay in nursing home for at least 3 months	Mean age 85.0 (7.7), 74% female IG *n* = 170 CG *n* = 152 Sweden *n* = 85 Norway *n* = 171 Denmark *n* = 66
Norway					
Langeland et al. (2019)^a^	To determine the effect of reablement in home-dwelling adults on daily activities, physical function, health-related quality of life and coping as a sense of coherence	Multi-centre CCT Follow-up 10 weeks, 6 and 12 months	Community care	• ≥ 18 years old • Experiencing functional decline • Not having terminal illness, cognitive impairment or in need for institution-based rehabilitation or nursing home placement	Mean age 78.4 (10.9), 69% female IG *n* = 707 CG *n* = 121
Tuntland et al. (2015)^a^	To determine the effect on self-perceived activity performance and satisfaction with performance, physical functioning, and health-related quality of life	RCT Follow-up 3 and 9 months	Community care	• ≥ 18 years old • Referred for home-based services based on their self-reported activity limitations • Not having terminal illness, cognitive impairment or in need of institution-based rehabilitation or nursing home placement	Mean age 79 (10.1), 69% female IG *n* = 31 CG *n* = 30
The Netherlands					
Henskens et al. (2017)	To determine the effect of Movement-oriented Restorative Care in preservation of ADL independence and quality of life in nursing home residents with dementia	CCT Follow-up 3, 6, 9 and 12 months	Institutionalised long-term care	• ≥ 65 years old • Diagnosis of dementia • Living in a psychogeriatric ward for at least 3 weeks • Not having bad vision, psychotic symptoms, very severe dementia, MMSE-score of ≥ 24 or medical contraindications for physical activities	Mean age 85.6 (6.2), 76% female IG *n* = 40 CG *n* = 26
Rooijackers et al. (2021)	To determine the effect of Stay Active at Home on sedentary behaviour in older homecare clients	RCT	Community care	• ≥ 65 years old • Receiving home care services • Not unable to communicate, having terminal illness, bed-bound mobility or having cognitive or psychological impairment	Mean age 82.1 (6.9), 67.8% female IG *n* = 133 CG *n* = 131

*RCT* randomised controlled trial, *CCT* clinical controlled trial, *MMSE* mini-mental state examination, *BI* Barthel Index, *IG* intervention group, *CG* control group

^a^Study effective on improving ADL functioning

### Outcomes and effects of reablement interventions on ADL functioning

Outcome measures, follow-up periods and outcome effects are shown in Table 2. ADL outcomes were measured with twelve different measures, of which ten were validated and/or standardised. Seventeen studies used validated and/or standardised outcome measures, while three studies used a self-developed non-standardised tool (Gitlin et al. 2006; Lewin et al. 2013; Lewin and Vandermeulen 2010). The most common measure, used in six studies, was the (unmodified) Barthel Index (Galik et al. 2014, 2015; Henskens et al. 2017; Powell et al. 2002; Resnick et al. 2011; Sackley et al. 2009). Six studies used a separate outcome measure concerning iADL (Gitlin et al. 2006, 2010; Lewin and Vandermeulen 2010; Rooijackers et al. 2021; Szanton et al. 2019; Tinetti et al. 2002). In five studies, ADL was a secondary outcome measure (King et al. 2012; Lewin et al. 2013; Parsons et al. 2020, 2017; Rooijackers et al. 2021). In general, the total study duration varied from 4 to 48 months. Within these periods, the first follow-up varied from 10 weeks to 6 months, and the second to fourth follow-ups varied from 6 to 12 months.

**Table 2:** Outcomes measures and effects on daily functioning of included studies (*n* = 20)

Author (year)	Outcome measure ADL^a^	Effect on ADL functioning	
		Follow-up	Results
United States of America			
Galik et al. (2014)	Barthel Index (range 0–100)	3 months	Significant improvement in mean scores in favour of intervention group: IG = 55.20 vs. CG = 44.32*
		6 months	No significant differences in mean scores between the groups
Galik et al. (2015)	Barthel Index (range 0–100)	3 months	No significant differences in mean scores between the groups
		6 months	No significant differences in mean scores between the groups
Gitlin et al. (2006)	Activities of Daily Living index (range 1–5 difficulty in previous month)	6 months	Significant improvement in mean scores in favour of intervention group: IG = 1.58 vs. CG = 1.66, with largest benefits occurring in bathing and toileting*
		12 months	Significant improvement was sustained, not specified*
	Instrumental Activities of Daily Living index (range 1–5 difficulty in previous month)	6 months	Significant improvement in mean score in intervention group, decline in control group: IG = 1.97 vs. CG = 2.07*
		12 months	Significant improvements were sustained, not specified*
Gitlin et al. (2010)	Functional Independence Measure—Overall functional dependence (lower score indicates higher dependency)	4 months	Significant improvement in favour of intervention group: IG = 3.7 vs. CG = 3.3, with adjusted mean difference 0.24*
		9 months	No significant differences in mean scores between the groups
	Subscale Activities of Daily Living dependence (lower score indicates higher dependency)	4 months	No significant differences in mean scores between the groups
		9 months	No significant differences in mean scores between the groups
	Subscale instrumental Activities of Daily Living dependence (lower score indicates higher dependency)	4 months	Significant improvement in mean scores in favour of intervention group: IG = 2.8 vs. CG = 2.5, with adjusted mean difference 0.32**
		9 months	No significant differences in mean scores between the groups
Resnick et al. (2011)	Barthel Index (range 0–100)	4 months	No significant differences in mean scores between the groups
		12 months	Significant decline in mean scores to the detriment of control group: IG = 69.19 vs. CG = 64.42**
Szanton et al. (2019)	ADL score (range 0–16)	5 months	Significant improvement in mean scores in favour of intervention group: IG = 2.22 vs. CG = 2.82** Treatment effect in favour of intervention group in RR (95% CI) 0.70 (0.54–0.93)**
		12 months	No significant differences in mean scores between the groups
	iADL score (range 0–16)	5 months	No significant differences in mean scores between the groups
		12 months	No significant differences in mean scores between the groups
Tinetti et al. (2002)	Self-care (range 0–12)	At completion of intervention (average 35 days)	Significant improvement in mean scores in favour of intervention group: IG = 11.0 vs. CG = 10.7*
	Home management (range 0–14)	At completion of intervention (average 35 days)	Significant improvement in mean scores in both groups: IG = 9.5 vs. CG = 9.2* Significant mean difference in both groups: IG = 5.8 vs. CG = 5.6*
New Zealand			
Kerse et al. (2008)	Late Life Function and Disability Instruments (lower score indicates higher dependency)	6 months	No significant differences in mean scores between the groups
		12 months	No significant differences in mean scores between the groups
King et al. (2012)	Nottingham Extended Activities of Daily Living—index (range 0–66)	4 months	No significant differences in mean scores between the groups
		7 months	No significant differences in mean scores between the groups
Parsons et al. (2017)	interRAI Home Care instrument (lower score indicates higher dependency)	Regular intervals over 24 months, not specified	No significant differences in mean scores between the groups
Parsons et al. (2020)	interRAI Contact Assessment (higher scores indicates higher dependency)	12 months	No significant differences in mean scores between the groups
Australia			
Lewin and Vandermeulen (2010)	Primary Assessment Form ADL (range 9–29)	3 months	Significant difference in change in favour of IG (Mann–Whitney *U* test) *z* = − 3.71**
		12 months	Significant difference in change in favour of IG (Mann–Whitney *U* test) *z* = − 2.90*
	Primary Assessment Form iADL (range 8–30)	3 months	Significant difference in change in favour of IG (Mann–Whitney *U* test) *z* = − 4.20**
		12 months	Significant difference in change in favour of IG (Mann–Whitney *U* test) *z* = − -3.24**
Lewin et al. (2013)	HACC Needs Identification (lower score, is higher dependency)	3 months	Significant improvement in mean scores in favour of intervention group, not specified* Significant improvement on independence of "showering": IG = 60% vs. CG = 23%**
		12 months	Significant improvement in mean scores was sustained, except iADLs were control group showed an increase in dependency, resulting in significant difference in favour of intervention group* Significant improvement on independence of "showering": IG = 58% vs. CG = 25%**
United Kingdom			
Powell et al. (2002)	Unmodified Barthel Index (range 0–20)	End of treatment (not specified)	Significant improvement in favour of intervention group: 35% vs. 20%* Median change score 0.00 due to ceiling effects
	The Functional Independence Measure and Functional Assessment Measure	End of treatment (not specified)	No significant differences in mean scores between the groups
Sackley et al. (2009)	Unmodified Barthel Index (range 0–20)	3 months	No significant differences in mean scores between the groups
		6 months	No significant differences in mean scores between the groups
Sweden, Norway and Denmark			
Gronstedt et al. (2013)	Functional Independence Measure, items a-m (lower score indicates higher dependency)	3 months	Significant decline in mean score in control group: CG = 42* No significant differences in mean scores between the groups
Norway			
Langeland et al. (2019)	Canadian Occupational Performance Measure—Performance (range 1–10)	10 weeks	Significant improvement in mean score in intervention group, decline in control group: IG = 3.19 (2.98, 3.40) vs. CG = 1.57 (1.12, 2.02))** Treatment effect mean difference in favour of IG (1.61 (1.13, 2.10))**
		6 months	Significant improvement and decline was sustained: IG = 3.19 (2.91, 3.46) vs. CG = 1.77 (1.21, 2.33)** Treatment effect mean difference in favour of IG (1.42 (0.82, 2.02))**
		12 months	No significant differences in mean scores between the groups. No significant treatment effect
	Canadian Occupational Performance Measure—Satisfaction (range 1–10)	10 weeks	Significant improvement in mean score in intervention group, decline in control group: IG = 3.43 (3.23, 3.64) vs. CG = 1.96 (1.50, 2.42)** Treatment effect mean difference in favour of IG (1.47 (0.98, 1.97))**
		6 months	Significant improvement in mean score in intervention group, decline in control group: IG = 3.41 (3.15, 3.67) vs. CG = 2.04 (1.48, 2.61)** Treatment effect mean difference in favour of IG (1.37 (0.77, 1.98))**
		12 months	No significant differences in mean scores between the groups. No significant treatment effect
Tuntland et al. (2015)	Canadian Occupational Performance Measure—Performance (range 1–10)	3 months	Significant improvement in mean scores in favour of intervention group: IG = 6.9 (6.1–7.8) vs. CG = 5.5 (4.7–6.3))*
		9 months	No significant differences in mean scores between the groups Overall treatment effect mean difference in favour of intervention group (1.5 (0.4–2.6))**
	Canadian Occupational Performance Measure—Satisfaction (range 1–10)	3 months	No significant differences in mean scores between the groups
		9 months	Significant decline in mean scores to the detriment of control group: IG = 6.5 (5.2–7.8) vs. CG = 5.2 (4.5–5.9)* Overall treatment effect mean difference in favour of intervention group (1.2 (0.1–2.3))*
The Netherlands			
Henskens et al. (2017)	Unmodified Barthel Index (range 0–20)	3 months	No significant differences in mean scores between the groups
		6 months	No significant differences in mean scores between the groups
		9 months	No significant differences in mean scores between the groups
		12 months	No significant differences in mean scores between the groups
Rooijackers et al. (2021)	Groningen Activiteiten Restrictie Schaal total score (range 18–72)	12 months	No significant differences in mean scores between the groups
	Groningen Activiteiten Restrictie Schaal ADL score (range 11–44)	12 months	No significant differences in mean scores between the groups
	Groningen Activiteiten Restrictie Schaal iADL score (range 7–28)	12 months	No significant differences in mean scores between the groups

*CG* control group, *IG* intervention group, *ADL* activities of daily living

^a^Activities of Daily Living in terms of basic ADL, instrumental ADL and advanced ADL

^b^Percentages are given due to ceiling effect at intake for 60% of participants, with 14% scoring 18 or 19 median change score is zero in both groups

Underlined scores indicate the most favourable scores: *p < 0.05; ^**^p ≤ 0.01

With regards to the effects, ten studies described a significant improvement in ADL functioning in terms of bADL and/or iADL at the first follow-up (Galik et al. 2014; Gitlin et al. 2006, 2010; Langeland et al. 2019; Lewin et al. 2013; Lewin and Vandermeulen 2010; Powell et al. 2002; Szanton et al. 2019; Tinetti et al. 2002; Tuntland et al. 2015). At the second follow-up, six studies showed either a significant improvement in favour of the intervention group (Langeland et al. 2019; Lewin et al. 2013; Lewin and Vandermeulen 2010; Powell et al. 2002; Tuntland et al. 2015) or improvements were sustained from the first follow-up (Gitlin et al. 2006). The studies that also measured iADL demonstrated significant improvements at the first follow-up in all except one study (Szanton et al. 2019). One study showed that improvements were sustained at the first follow-up (Gitlin et al. 2006), and another study showed significant treatment effects in favour of the intervention group (Lewin and Vandermeulen 2010).

### Intervention characteristics

To identify common features of the included interventions, the following results discuss the features that the interventions (*n* = 20) had most in common in terms of setting, duration, intensity, team composition, staff training, target group and the different intervention components. All interventions used a person-centred and holistic approach. These intervention characteristics and content, based on the criteria of the ReAble definition, are shown in Table 3.

**Table 3:** Intervention components of included reablement intervention studies (*n* = 20)

Author (year)	Intervention aim	Characteristics			Components		
		Duration and intensity	Interdisciplinary team	Coordination and collaboration method	Initial comprehensive assessment and regular reassessments	Goal-oriented support plan	Interventions to reach clients' goals
United States of America							
Galik et al. (2014)^a^	To promote nursing care on optimising physical activity in residents	Duration and intensity not specified	Nursing assistants, FFCC FFCN, nursing home staff not specified	Coordination and collaboration method not specified	Not specified	Assessment by FFCN and input from family, staff, and FFCC	• ADL training • Education • Physical and/or functional exercise
Galik et al. (2015)	To promote residents to participate in functional activities and be physically active	Duration and intensity not specified	Direct care workers, FFCC, FFCN	Coordination and collaboration method not specified	Not specified	Assisted Living Resident Assessment Form, therapy notes, range of motion and cognition by FFCN and FFCC with input from direct care workers and families	• ADL training • Adaptations • Education
Gitlin et al. (2006)^a^	To compensate for declining abilities	Maximum of 24 weeks 5 OT sessions of 20–90 min, 1 PT session of 90 min	OT and PT	Coordination between OT and PT for integrated approach, not specified	Semi-structured clinical interview by OT Reassessment not specified	Semi-structured clinical interview by OT	• ADL training • Adaptations • Education •Physical and/or functional exercise
Gitlin et al. (2010)^a^	To decrease sensorial, physical, and cognitive demands, promote patients in daily activities, and increase functionality, alleviating caregiver burden	Maximum of 16 weeks On average 8—12 sessions, 68.24 min face-to-face, and telephone sessions of 20.15 min by OT, and 6.27 min by nurses	OT, advanced practice nurse, family caregiver	Coordination and collaboration method not specified	Interview of caregivers and test of cognitive and functional capabilities of client by OT Reassessment not specified	Written action plan describing treatment goals, patient strengths, and specific strategies by OT	• ADL training • Adaptations • Education • Physical and/or functional exercise • Functional disorder management
Resnick et al. (2011)	To change how direct care workers provide care to maintain and improve function and physical activity	Duration and intensity not specified	Direct care worker, FFCC, FFCN	Coordination and collaboration method not specified	Not specified	Goal Attainment Forms by FFCN, with input from FFCC and staff	• ADL training • Adaptations • Education • Physical and/or functional exercise
Szanton et al. (2019)^a^	To improve daily function and meet the needs of low-income older adults	Maximum 20 weeks On average 8—10 sessions, OT ≤ 6 sessions for ≤ 1 h, an RN ≤ 4 sessions for ≤ 1 h)	OT, RN, handyman	Coordination by OT for case-coordination with other interventionists Collaboration method between the OT, nurse, and handyman through a secure share site	Client–Clinician Assessment Protocol by OT and RN Reassessment at 20 weeks	Goal setting by OT and RN, not specified	• ADL training • Adaptations • Education • Physical and/or functional exercise • Functional disorder management
Tinetti et al. (2002)^a^	To determine effect on functional status, remaining at home, duration and intensity of home care episode, emergency visits, and pain or dyspnoea	On average 5 weeks On average 12.5 sessions of PT (2.1), nurses (6.8) and home health aides (3.1)	Home care nurses, OT, PT, home health aides	Coordination and collaboration method not specified	OASIS-B standardized assessment by home care staff Reassessment not specified	Goal setting by home care staff with input from family, not specified	• ADL training • Adaptations • Education • Physical and/or functional exercise • Functional disorder management
New Zealand							
Kerse et al. (2008)	To promote independence in residential care	52 weeks Up to 30 min/day for more dependent residents	Gerontology nurse, nursing assistant PT and OT available on demand	Coordination by gerontology nurse, assisted by PT and OT if needed Collaboration method not specified	Functional assessment by gerontology nurse with assistance of OT or PT if needed Reassessment not further specified	Goal setting by gerontology nurse, not specified	• ADL training • Physical and/or functional exercise
King et al. (2012)	To reduce need for long-term support and promote quality of home care services	Duration and intensity not specified	Paid caregivers, coordinator (experienced RN) Referrals to OT, PT, meal preparation etc	Coordination by RN Collaboration method not specified	TARGET by coordinator Reassessment at 12 months	TARGET by coordinator	• ADL training • Functional disorder management
Parsons et al. (2017)	To meet the needs of high-risk older people at home	Duration and intensity not specified	RN, healthcare assistants	Coordination by RN for case management •Daily contact with non-regulated support workers •Clinical (assessment etc.) and non-clinical (supervision etc.) duties Collaboration method not specified	interRAI Home Care Assessment (complex care) by locality-based health professionals interRAI Contact Assessment (non-complex care) by home care services health professional coordinators Reassessment every 12 weeks	Comprehensive geriatric assessment and "goal ladder" by RN case manager coordinator	• ADL training • Physical and/or functional exercise
Parsons et al. (2020)	To promote independence rather than foster dependence	Maximum of 6 weeks Intensity not specified	OT, PT, RNs, healthcare assistants, consultant geriatricians	Coordination not specified to one person: RNs, OTs, and PTs responsible to intervention Collaboration method by weekly team case conferences and collaboration with patient's community, care team, and hospital services	InterRAI Contact Assessment by RN/healthcare assistant Reassessment not further specified	Goal facilitation tool by RN/healthcare assistant, not further specified	• ADL training • Physical and/or functional exercise
Australia							
Lewin and Vandermeulen (2010)^a^	To promote functioning, preventing or delaying functional decline, promoting healthy ageing and encouraging the self-management of chronic diseases	Maximum of 12 weeks or until they achieve their goals, whichever is sooner, on average 9 weeks Intensity not specified	OT, PT, RN, care manager Other caregivers available on demand	Coordination by care manager Collaboration method by weekly HIP care team meetings	Comprehensive multi-dimensional assessment (MDA) by care manager Reassessment at 12 weeks	HACC Needs Identification (HNI) within MDA by care manager	• ADL training • Education • Physical and/or functional exercise • Functional disorder management
Lewin et al. (2013)^a^	To promote functioning, preventing or delaying functional decline, promoting healthy ageing and encouraging the self-management of chronic diseases	Maximum of 12 weeks or until they achieve their goals, whichever occurs first Intensity not specified	OT, PT, RN, care manager (interdisciplinary team member) Other caregivers available on demand	Coordination by care manager Collaboration method by weekly HIP care team meetings	Comprehensive multi-dimensional assessment by care manager Reassessment at 12 weeks	Goal-oriented care planning by care manager, not specified	• ADL training • Education • Physical and/or functional exercise • Functional disorder management
United Kingdom							
Powell et al. (2002)^a^	To promote independence in ADL, social participation, and psychological wellbeing	Maximum of 60 weeks, on average 28 weeks On average 2–6 h per week	OT, PT, speech and language therapist, clinical psychologist, social worker	Coordination and collaboration method not specified	Functional Assessment Measure (FAM) by two therapists Reassessment at end of treatment	“Contractually organised goal setting” by carer and team	• ADL training • Physical and/or functional exercise
Sackley et al. (2009)	To promote independent living and mobility among care home residents over and above that achieved with standard care	Maximum of 12 weeks On average 6.4 sessions PT and 132.6 min contact, on average 9.8 sessions OT and 216 min contact	PT, OT, home care staff	Coordination and collaboration method not specified	Assessment physical and functional status and equipment needs by PT and OT Reassessment not further specified	Functional goal setting by PT and OT, not specified	• ADL training • Adaptations • Physical and/or functional exercise
Sweden, Norway, Denmark							
Gronstedt et al. (2013)	To prevent unnecessary functional decline	On average 10–13 weeks On average 117 min/week	OT, PT, nursing home staff	Coordination by OT and PT, also providing guidance of staff Collaboration method not specified	Clinical assessment by OT and PT Reassessment not further specified	Listing important activities by OT and PT, not specified	• ADL training • Adaptations • Education • Physical and/or functional exercise
Norway							
Langeland et al. (2019)^a^	To achieve the activity goals set in the interdisciplinary assessment	Maximum of 10 weeks, on average 6 weeks On average 3–5 sessions a week	OT, PT, (auxiliary) nurse, and home helper	Coordination not specified to one person: collaboration interdisciplinary team with participant Collaboration method not specified	Canadian Occupational Performance Measure by OT/PT/Nurse Reassessment at 10 weeks	Canadian Occupational Performance Measure by OT/PT/Nurse	• ADL training • Adaptations • Education • Physical and/or functional exercise
Tuntland et al. (2015)^a^	To promote independence in ADL, enable people to age in place, be active and participate socially in society	Maximum of 12 weeks, on average 10 weeks On average 6.5 sessions/1.9 h per week	OT, PT, (auxiliary) nurses, social educators, home helpers and assistants	Coordination not specified Collaboration method by weekly informal meetings	Canadian Occupational Performance Measure by OT/PT Reassessment at end 10 weeks	Canadian Occupational Performance Measure by OT/PT	• ADL training • Adaptations • Education • Physical and/or functional exercise
The Netherlands							
Henskens et al. (2017)	To optimise independence in ADL and QoL	Maximum of 52 weeks	Nursing staff, department heads, PT, OT, psychologists, geriatricians, and activity supervisors, volunteers and family members	Coordination not specified Collaboration method by staff meetings, not specified	Selection and evaluation of functional capabilities by primary responsible caregiver Reassessment not specified	Goal setting by nursing staff, not specified	• ADL training • Education • Physical and/or functional exercise
Rooijackers et al. (2021)	To reduce clients' sedentary behaviour and promote independence	Duration and intensity not specified	RN, nurse assistants, nurse aides and domestic support workers	Coordination by district nurse Collaboration method not specified	No assessment tool used by nursing staff Reassessment not specified	Goal setting by nursing staff using the SMART-method	• ADL training • Education • Physical and/or functional exercise

*FFCC* function-focused care champion, FFCN function-focused care nurse, OT occupational therapist, PT physical therapist, RN registered nurse, ADL activities of daily living, QoL quality of life, SMART specific, measurable, attainable, relevant, time-bound

^a^Study effective on improving ADL functioning

#### Setting

In thirteen interventions, the setting was the participant's home (Gitlin et al. 2006, 2010; King et al. 2012; Langeland et al. 2019; Lewin et al. 2013; Lewin and Vandermeulen 2010; Parsons et al. 2020, 2017; Powell et al. 2002; Rooijackers et al. 2021; Szanton et al. 2019; Tinetti et al. 2002; Tuntland et al. 2015), while in seven interventions, the setting was a long-term care facility (Galik et al. 2014, 2015; Gronstedt et al. 2013; Henskens et al. 2017; Kerse et al. 2008; Resnick et al. 2011; Sackley et al. 2009).

#### Intervention duration and intensity

Thirteen interventions were time-limited and had a mean duration of 15.7 weeks (range 5–60) (Gitlin et al. 2006, 2010; Gronstedt et al. 2013; Henskens et al. 2017; Kerse et al. 2008; Langeland et al. 2019; Lewin et al. 2013; Lewin and Vandermeulen 2010; Parsons et al. 2020; Powell et al. 2002; Sackley et al. 2009; Szanton et al. 2019; Tuntland et al. 2015). In two studies, the intervention could also end earlier, when participants had achieved their set goals (Lewin et al. 2013; Lewin and Vandermeulen 2010). Seven studies did not specify a maximum duration (Galik et al. 2014, 2015; King et al. 2012; Parsons et al. 2017; Resnick et al. 2011; Rooijackers et al. 2021; Tinetti et al. 2002), in addition, ten studies specified the intensity of the intervention, in terms of the amount and duration of sessions given (Gitlin et al. 2006, 2010; Gronstedt et al. 2013; Kerse et al. 2008; Langeland et al. 2019; Powell et al. 2002; Sackley et al. 2009; Szanton et al. 2019; Tinetti et al. 2002; Tuntland et al. 2015). Sessions varied from one session every four weeks to three sessions per week, with an average duration ranging from 0.1 to 6 h per week. On average, the effective interventions had a slightly shorter duration compared to non-effective interventions (mean 15.4 weeks, range 5–60 vs. mean 17.5 weeks, range 6–52). The intensity in terms of sessions and minutes spent per week could not be compared due to a lack of intervention details.

#### Interdisciplinary teams

Most interdisciplinary teams consisted of registered nurses (RN), nursing assistants (NA), occupational therapists (OT) and physical therapists (PT). In two studies, a medical specialist was also involved (Henskens et al. 2017; Parsons et al. 2017). On average, effective interventions had a more diverse team of health care professionals as a part of their interdisciplinary team than non-effective interventions (mean 3.5, range 2–6 vs. mean 3.2, range 2–7). In addition, results showed that OTs and PTs were a standard part of the team in seven of the effective interventions (Gitlin et al. 2006; Langeland et al. 2019; Lewin et al. 2013; Lewin and Vandermeulen 2010; Powell et al. 2002; Tinetti et al. 2002), in contrast to four of the non-effective interventions (Gronstedt et al. 2013; Kerse et al. 2008; King et al. 2012; Parsons et al. 2020; Sackley et al. 2009).

All studies described a coordinated collaboration between care professionals, only the method of collaboration and communication was not always specified. Four studies reported details on the frequency of these team meetings, which took place weekly (Langeland et al. 2019; Lewin et al. 2013; Lewin and Vandermeulen 2010; Parsons et al. 2020). Five effective interventions described that a member of the interdisciplinary team coordinated the intervention; this was often an RN, OT, or PT (Gitlin et al. 2006; Langeland et al. 2019; Lewin et al. 2013; Lewin and Vandermeulen 2010; Szanton et al. 2019), other effective interventions did not provide further details on the coordinator. Six of the non-effective interventions described that a registered nurse was appointed as the intervention coordinator (Kerse et al. 2008; King et al. 2012; Parsons et al. 2020, 2017; Resnick et al. 2011; Rooijackers et al. 2021), other non-effective interventions did not provide further details on the coordinator. The coordinators took the initial assessment and reassessment, set out goals with participants, monitored progress and coordinated team meetings and the education of staff.

#### Training of the interdisciplinary teams

In all but two studies (Parsons et al. 2020; Powell et al. 2002), staff training was described. Staff received specific training regarding care delivery (e.g. goal-setting tools, assessment procedures, etc.) in the form of lectures, seminars, courses, and education by other members of the team. This training varied in duration from one day to the form of weekly educational meetings over the intervention period varying from 5 to 60 weeks. Staff training could not be compared between effective and non-effective interventions due to a lack of detail regarding the contents of the training sessions given.

#### Target group

The thirteen studies conducted in community care described their target group in general as individuals in need of home care services or experiencing a functional decline. All interventions were aimed at individuals of ≥ 60 years old, except for three interventions that also included individuals of ≥ 18 years (Gitlin et al. 2010; Langeland et al. 2019; Tuntland et al. 2015), and one intervention including individuals between 16 and 65 years old (Powell et al. 2002). In terms of participant capacity, all interventions were aimed at individuals that required assistance with one or more ADLs, and/or experienced functional decline but were not completely care-dependent. One intervention was not only aimed at individuals with a diagnosis of dementia but also their caregivers (Gitlin et al. 2010); another was specifically aimed at individuals with Traumatic Brain Injury (Powell et al. 2002). The eighteen other interventions specifically excluded individuals in case of terminal illness, neurological disorder, and diagnosis of dementia because, according to the authors of these studies, these groups would benefit the least from the intervention or would eventually require institution-based rehabilitation or nursing home placement. Because only four non-effective interventions took place in community care (King et al. 2012; Parsons et al. 2020, 2017; Rooijackers et al. 2021), the target group cannot be compared with the target groups of the fifteen effective interventions that took place in community care.

The seven studies conducted in institutionalised long-term care described the target group as residents with a minimum expected stay of 3 months, and a minimum age of 55 years old. Three interventions were specifically aimed at individuals with a diagnosis of dementia (Galik et al. 2014, 2015; Henskens et al. 2017). Regarding the participants' capacity, all interventions were aimed at individuals needing assistance in their ADLs, and not meant for individuals in rehabilitation or cases of terminal care. As only one effective intervention that took place in institutionalised long-term care showed effects (Galik et al. 2014), the target group could not be compared with the target groups of the non-effective interventions.

### Intervention components

#### Initial comprehensive assessment and regular reassessments

The assessment used in all interventions was generally interdisciplinary. A standardised instrument was used in ten interventions (King et al. 2012; Langeland et al. 2019; Lewin et al. 2013; Lewin and Vandermeulen 2010; Parsons et al. 2020, 2017; Powell et al. 2002; Szanton et al. 2019; Tinetti et al. 2002; Tuntland et al. 2015), for example, the interRAI Home Care Assessment (Landi et al. 2000). Six interventions used either a semi-structured interview method or a profession-specific intake assessment (Gitlin et al. 2006, 2010; Gronstedt et al. 2013; Henskens et al. 2017; Kerse et al. 2008; King et al. 2012) and four interventions did not use a standardised or protocoled assessment (Galik et al. 2014, 2015; Resnick et al. 2011; Rooijackers et al. 2021). Reassessments took place at intervals ranging from 10 to 52 weeks in seven interventions (King et al. 2012; Langeland et al. 2019; Lewin et al. 2013; Lewin and Vandermeulen 2010; Parsons et al. 2017; Szanton et al. 2019; Tuntland et al. 2015). The other interventions did not specify either if or when the reassessment took place. The results show that in seven effective interventions (Langeland et al. 2019; Lewin et al. 2013; Lewin and Vandermeulen 2010; Powell et al. 2002; Szanton et al. 2019; Tinetti et al. 2002; Tuntland et al. 2015), a standardised or protocoled assessment method was used, whereas this was the case in three of the non-effective interventions (King et al. 2012; Parsons et al. 2020, 2017).

#### Goal-oriented support plan

In sixteen interventions, the assessment method was also used to identify activities the participant perceived as meaningful and to develop a person-centred goal-oriented support plan (Gitlin et al. 2006, 2010; Gronstedt et al. 2013; Henskens et al. 2017; Kerse et al. 2008; King et al. 2012; Langeland et al. 2019; Lewin et al. 2013; Lewin and Vandermeulen 2010; Parsons et al. 2020, 2017; Powell et al. 2002; Sackley et al. 2009; Szanton et al. 2019; Tinetti et al. 2002; Tuntland et al. 2015). Four effective interventions and one non-effective intervention used validated and standardised goal-setting instruments (King et al. 2012; Langeland et al. 2019; Lewin et al. 2013; Lewin and Vandermeulen 2010; Tuntland et al. 2015), for example, the Canadian Occupational Performance Measure (Law et al. 1990). In the other interventions, semi-structured interviews, the SMART method (Day and Tosey 2011), lists of important activities for the participant or input from other healthcare professionals and their families were used to set goals.

#### Interventions to reach clients’ goals

Five intervention components were identified. Effective interventions contained a broader offer of intervention components to reach clients’ goals than non-effective interventions (mean 4, range 2–5 vs. mean 2.8, range 2–4). ADL training, physical and/or functional exercise and education were the most common, and functional disorder management the least common components. Environmental adaptations were also an identified intervention component but did not recur as often as the other four. The specifications of these interventions are as follows.

First, ADL training was a recurring component in all effective and non-effective interventions. This entailed individual training in activities such as personal care and eating by encouraging clients to do (a part of) the activity themselves, followed by repeating and incorporating this training within the clients’ daily life. The participant also learned strategies to adapt an activity and make use of helping aids. OTs or RNs usually gave ADL training. No statements about effectiveness can be made because the same amount of effective and non-effective interventions included ADL training.

Second, physical and/or functional exercise was also a recurring component in all effective and non-effective interventions. Physical and/or functional exercises focused on physical activity in the training of strength, balance, endurance, and fine motor skills, but also on promoting active engagement in social and group activities. Training took place both individually as well as in group sessions. In most cases, it was not specified who provided the exercises, but if they did, it was often the PT who offered them. No statements about effectiveness can be made because the same amount of effective and non-effective interventions included physical and/or functional exercise.

Third, education played a role in reaching clients' goals in nine out of ten effective interventions (Galik et al. 2014; Gitlin et al. 2006, 2010; Langeland et al. 2019; Lewin et al. 2013; Lewin and Vandermeulen 2010; Szanton et al. 2019; Tinetti et al. 2002; Tuntland et al. 2015) and five non-effective interventions (Galik et al. 2015; Gronstedt et al. 2013; Henskens et al. 2017; Resnick et al. 2011; Rooijackers et al. 2021). Participants were educated on self-management, building confidence, healthy ageing, problem-solving, prevention strategies, stimulating (physical) activity and medication use. In addition to educating the participants, five effective interventions specifically described family and/or caregivers also receiving education to stimulate the participant in becoming less dependent on care (Galik et al. 2014; Gitlin et al. 2010; Lewin et al. 2013; Lewin and Vandermeulen 2010; Tinetti et al. 2002), this was also the case in one non-effective intervention (Galik et al. 2015). It was often not specified which member of the interdisciplinary team provided education.

Fourth, environmental adaptations played a role in six effective interventions (Gitlin et al. 2006, 2010; Langeland et al. 2019; Szanton et al. 2019; Tinetti et al. 2002; Tuntland et al. 2015) and four non-effective interventions (Galik et al. 2015; Gronstedt et al. 2013; Resnick et al. 2011; Sackley et al. 2009). Adaptations mentioned were the use of assistive technology (e.g. walking aids), home environment adaptations or repairs (e.g. safety rails), and providing medical equipment (e.g. blood pressure monitor). An OT often provided adaptations.

Last, the management of functional disorders, such as pain, continence, nutrition, skin integrity, testing of blood and urine, and management of medication, was incorporated in five effective interventions (Gitlin et al. 2010; Lewin et al. 2013; Lewin and Vandermeulen 2010; Szanton et al. 2019; Tinetti et al. 2002) and one non-effective intervention (King et al. 2012). The coordinator or RN involved often provided functional disorder management.

### Risk of bias

Findings on the risk of bias are shown in Table 4. The RCTs scored 62% (range 38–92) on average on the JBI Critical Appraisal Checklist for Randomised Controlled Trials (Joanna Briggs Institute 2017b), and the CCTs 53% (range 44–56) on the JBI Checklist for Quasi-Experimental (Joanna Briggs Institute 2017a). Four of the twenty studies were judged at low risk of bias (Gitlin et al. 2006; Parsons et al. 2020; Szanton et al. 2019; Tuntland et al. 2015), eleven at moderate risk, and four at high risk (Galik et al. 2015; Lewin et al. 2013; Resnick et al. 2011; Tinetti et al. 2002). No RCT was able to blind participants and delivery personnel to treatment assignment. In seven studies treatment groups were similar at baseline (Galik et al. 2014; Gitlin et al. 2006; Gronstedt et al. 2013; Parsons et al. 2020; Powell et al. 2002; Rooijackers et al. 2021; Tuntland et al. 2015). Risk of bias assessment demonstrated that in all CCTs the effect could also be explained by other exposures or treatments occurring at the same time. In most studies, follow-up was not adequately described and analysed and lacked appropriate statistical analysis. The latter was mainly due to a lack of power.

**Table 4:** Risk of bias assessment of included randomised controlled trials (*n* = 16) and clinical controlled trials (*n* = 4)

RCTs	Q1	Q2	Q3	Q4	Q5	Q6	Q7	Q8	Q9	Q10	Q11	Q12	Q13	% yes	Risk^a^
Galik et al. (2014)^b^	0	0	1	0	0	1	1	0	1	1	1	0	1	54	Moderate
Galik et al. (2015)	0	0	0	0	0	0	1	0	1	1	1	0	1	38	High
Gitlin et al. (2006)^b^	1	1	1	0	0	1	1	1	1	1	1	1	1	85	Low
Gitlin et al. (2010)^b^	1	1	0	0	0	0	1	1	1	1	1	1	1	69	Moderate
Resnick et al. (2011)	0	0	0	0	0	0	1	0	1	1	1	0	1	38	High
Szanton et al. (2019)^b^	1	1	0	0	0	1	1	1	1	1	1	1	1	77	Low
Kerse et al. (2008)	1	1	0	0	0	1	1	0	0	1	1	1	1	62	Moderate
King et al. (2012)	1	1	0	0	0	1	1	0	1	1	1	0	1	62	Moderate
Parsons et al. (2017)	1	1	0	0	0	0	1	0	1	1	1	1	1	62	Moderate
Parsons et al. (2020)	1	1	1	0	0	1	1	0	1	1	1	1	1	85	Low
Lewin et al. (2013)^b^	0	0	0	0	0	1	1	0	0	1	1	1	1	46	High
Powell et al. (2002)^b^	1	1	1	0	0	1	1	0	1	0	1	0	1	62	Moderate
Sackley et al. (2009)	1	1	0	0	0	1	1	1	1	1	1	0	1	69	Moderate
Gronstedt et al. (2013)	1	1	1	0	0	1	1	0	1	1	1	0	1	69	Moderate
Tuntland et al. (2015)^b^	1	1	1	0	0	1	1	1	1	1	1	1	1	85	Low
Rooijackers et al. (2021)	1	1	1	0	0	0	1	1	0	1	1	1	1	69	Moderate

CCTs	Q1	Q2	Q3	Q4	Q5	Q6	Q7	Q8	Q9	% yes	Risk^a^
Tinetti et al. (2002)^b^	1	0	0	1	1	0	0	1	0	44	High
Lewin and Vandermeulen (2010)^b^	1	0	0	1	1	0	1	1	0	56	Moderate
Langeland et al. (2019)^b^	1	0	0	1	1	0	1	1	0	56	Moderate
Henskens et al. (2017)	1	0	0	1	1	1	0	1	0	56	Moderate

*Note* Risk of Bias was assessed using the Joanna Briggs Institute Critical Appraisal Checklist for Randomised Controlled Trials and the Checklist for Quasi-Experimental Studies (non-randomised experimental studies) (Joanna Briggs Institute, 2017a, b)

*JBI* Joanna Briggs Institute, *RCT* randomised controlled trial, *CCT* clinical controlled trial

^a^Risk of bias ranked as high when percentage up to 49%, moderate when percentage from 50 to 69%, and a low percentage of more than 70% of “yes” scores. 1 indicates yes, 0 indicates unclear or no

^b^Study effective on improving ADL functioning

Overall, within the effective interventions, three studies scored low (Gitlin et al. 2006; Szanton et al. 2019; Tuntland et al. 2015), five scored moderate (Galik et al. 2014; Gitlin et al. 2010; Langeland et al. 2019; Lewin and Vandermeulen 2010; Powell et al. 2002) and two scored high on the risk of bias assessment (Lewin et al. 2013; Tinetti et al. 2002). Within the non-effective interventions, one study scored low (Parsons et al. 2020), seven moderate (Gronstedt et al. 2013; Henskens et al. 2017; Kerse et al. 2008; King et al. 2012; Parsons et al. 2017; Rooijackers et al. 2021; Sackley et al. 2009) and two high on the risk of bias assessment (Galik et al. 2015; Resnick et al. 2011).

## Discussion

Using the ReAble definition as a starting point, this systematic review aimed to provide a current overview of reablement interventions internationally and their effect on clients’ daily functioning. Twenty relevant studies from eight countries were included in this systematic review. Ten of these studies were effective in improving ADL functioning. In addition, we intended to identify the most common and possibly promising features in an attempt to "unpack" existing reablement interventions. However, the identification of the most promising features was challenging as an equal amount of effective and non-effective interventions were identified and intervention content was poorly described. Nevertheless, there are some indications that interdisciplinary teams with more diverse disciplines, the use of standardised assessment/goal-setting instruments and four recurring intervention components (i.e. ADL-training, physical and/ or functional exercise, education, management of functional disorders) positively influence the effectiveness of reablement concerning clients’ daily functioning.

Concerning the outcome ADL functioning, we see a great variation in outcome measures used. Roughly, they can be divided into two groups: ADL measures that are goal-oriented and tailored to the individual (e.g. COPM), and more generic ADL measures (e.g. Barthel Index). These generic measures are less sensitive to detect small changes, which is essential when establishing minor improvements in independence (Hartigan 2007). However, combining more subjective measures, like the COPM, with more generic measures, such as the Barthel Index, helps to place the subjective assessment of ADL functioning in the right context (Mlinac and Feng 2016). Unfortunately, in this systematic review it is not possible to conclude how the chosen ADL outcomes have influenced the study findings.

As for factors impacting the effectiveness of reablement, the included studies show that both the size and composition of the interdisciplinary team may influence the effectiveness, with a more diverse team showing more often positive outcomes regarding daily functioning. These positive effects might be explained by the fact that, within a more diverse interdisciplinary team, there is a much broader base of knowledge, skills and resources available, allowing the problem to be approached from more different perspectives (Phillips and O'Reilly 1998). However, with increasing team size, the complexity of interdisciplinary collaboration also increases, especially when it comes to dividing tasks and responsibilities, which makes coordination of teams of utmost importance (Schmutz et al. 2019). In two-thirds of the included interventions, we see that often the RN, OT, and PT are a standard part of the reablement team. These disciplines could be valuable to include in a reablement team due to their educational background. For example, goal setting is part of their curricula and they often have interdisciplinary training, which has shown to contribute towards better collaborative skills and attitudes with other healthcare professions (Reeves et al. 2016; Rossler et al. 2017). Although, due to the differences in educational background, team members also have different values, beliefs, attitudes and behaviours, which emphasises the importance of training in teamwork (Schmutz et al. 2019). Other factors that should be taken into account concerning interdisciplinary collaboration are team familiarity, team members' experience, task complexity and time pressure (Schmutz et al. 2019). Close cooperation and evaluation, as well as time for communication, shared planning and decision making, and goal setting by the client contribute positively to interdisciplinary cooperation (Birkeland et al. 2017).

All included studies used an assessment/goal-setting instrument, as goal setting plays an essential role within reablement. Goal setting involves the client in the decision-making process and ensures that the client's values, autonomy and preferences are respected (Levack 2009; Munthe et al. 2012). However, care professionals often indicate that they lack the knowledge and skills to involve clients in goal setting, that clients are sometimes difficult to motivate, remain passive in the recovery process, and feel overwhelmed to take control of their rehabilitation (Rose et al. 2017). Based on our systematic review there are indications that the use of a standardised assessment/goal-setting tool may lead to more positive outcomes regarding daily functioning. Also, Rose et al. (2017) advise using standardised goal-setting tools to better involve clients in the process (Rose et al. 2017), such as TARGET (Parsons and Parsons 2012) and COPM (Law et al. 1990), which both facilitate the professional to identify activities meaningful to the client and set goals correspondingly. Nevertheless, it remains challenging to set good person-centred goals. To improve goal setting and shared decision, it is also recommended to train both health professionals and clients in shared decision making and the associated communication skills (Rose et al. 2017).

Regarding promising intervention components, it seems like effective interventions contained on average more diverse components. Complex interventions have gained increasing attention over the last years, but their content is often briefly reported or unknown, which makes it difficult to assess their effectiveness and to determine why they work or fail (Smit et al. 2018). Ideally, the modelling and processes of outcomes of complex interventions should be described to identify why the intervention works or does not work (Richards and Hallberg 2015; Smit et al. 2018). This is necessary to understand underlying mechanisms within and between the different intervention components and to properly determine their effectiveness. As addressed earlier, we also had to deal with poor intervention descriptions in many studies. Nevertheless, four possibly promising intervention components were identified.

First, ADL training is a recurring intervention component, which is included in all identified reablement programmes. Within the occupational therapy literature, great benefits are found when ADL training in the elderly population is carried out in their home environment (Liu et al. 2018). The use of home visits or home assessments is recommended to identify problems between the individual's capabilities and the environment. Based on this home assessment, interventions and evaluations can be more tailored and thus increase effectiveness (Liu et al. 2018).

Second, physical and/or functional exercises were also included in each reablement intervention. Mjøsund et al. (2020) studied how physical activity is integrated within reablement programmes. They found that most client goals were set in terms of functional mobility (such as walking, climbing stairs, outdoor walking). These exercises often focus on strength, balance or endurance, but specific details of the programmes were lacking. Liu et al (2018) found that physical exercise is often integrated into ADL training. When exercises are functional, task-specific, and meet client's wishes, they have more favourable outcomes on ADL performance than when they are more structured, constructive and repetitive. (Liu et al. 2018). According to the review by Blankevoort et al. (2010) it is recommended to combine different exercises such as strength, endurance and balance training to improve progress in physical functioning and performance in ADL rather than only providing progressive resistance training. The best results were achieved with the highest training volume. These results are also confirmed by the review by Theou et al. (2011) who looked at managing frailty in older people through exercise. The reviews both emphasise that exercise programmes that last longer than 12 weeks with an intensity of 3 times a week and sessions of 30–45 min produce the best results in functional, physical and psychosocial terms, and help prevent adverse health effects.

Third, education is regularly integrated into (effective) reablement programmes targeting the client and/ or their informal carers. On the one hand, education was given during educational meetings making use of handouts and leaflets, for example on how to motivate clients in daily and physical activities. On the other hand, advice was given by care professionals during regular care moments. Topics of education were, for example, on how to carry out (i) ADL activities, use of (mobility)aids and self-management.

Additionally, management of functional disorders, which is often provided by nurses, was included in five out of six effective interventions. The literature on essential nursing care (Kitson et al. 2010; Kitson and Muntlin Athlin 2013) emphasises the importance of care activities like eating and drinking, comfort (including pain management), safety, prevention and medication. However, this field of nursing care is often overlooked, undervalued and taken for granted, which can have a negative impact on client outcomes (Zwakhalen et al. 2018).

### Strengths and limitations

One of the strengths of this review is the process of obtaining the final study selection as a) the entire screening process was conducted independently by two reviewers, b) the final sample was checked and supplemented by experts with a broader view than only geriatrics, c) snowball sampling was used to reduce the risk of missing possible important articles, and d) the search was repeated after one year as the review should be as up to date as possible according to the methodological standards of the Cochrane Collaboration (Chandler et al. 2013). Moreover, grey literature was used to supplement the extracted data when available. The risk of bias assessment is another strength, as the reviewers completed the JBI checklists independently and reached a consensus through discussion in case this was necessary. Our systematic review focused on ADL functioning. Consequently, promising reablement features concerning other outcomes such as physical functioning, quality of life and fewer hospital admissions were not taken into account. Another limitation is that conclusions cannot be confidently drawn, as more than three-quarters of the included studies were of moderate to poor quality which hindered us to exclude the lower quality studies.

### Methodological reflection

Reflecting on the capability of the used research design in answering the research questions, this review has been able to provide an overview of current evidence and reflect on the effects on ADL functioning. While we have been able to identify (promising) components of the programmes, we do not yet know much about the details of these components in practice and whether they were implemented as intended. Since the latter is not usually discussed within effect studies, including results of process evaluations could be potentially valuable. In addition, it is suggested to conduct multiple case studies to gather more in-depth information about existing reablement programmes.

## Conclusions and practical implications

This study has several important implications for future practice regarding reablement interventions. First, reablement interventions should be delivered by a diverse interdisciplinary team, preferably including nurses, occupational therapists and/ or physical therapists, while attention should be paid to the training and coordination of the team and other factors that influence the quality of interdisciplinary collaboration. Second, reablement interventions should make use of standardised assessment and goal-setting tools, which should be combined with training for both healthcare professionals and clients. Third, promising intervention components are (i) ADL-training, physical and/or functional exercise, education and management of functional disorders.

A start has been made with 'unpacking' reablement; however, the review has only scratched the surface in terms of a better understanding of the determining factors for the effectiveness of reablement interventions. More research is needed to open the black box of reablement. First, more intervention protocols should be published that make use of reporting guidelines such as the TIDieR checklist (Hoffmann et al. 2014) and the use of process evaluations should be emphasised to assess the variation in results of effect studies within the right context (Moore et al. 2015). Second, collecting additional data from reablement experts, who have developed, evaluated and implemented reablement interventions, can provide more in-depth information about available reablement interventions. Third, more high-quality studies using outcomes tailored to the client's goals (e.g. COPM) are needed that aim to identify reablement features that are more promising than others and investigate which combination of features is most effective.

## Supplementary Information

Below is the link to the electronic supplementary material.

Supplementary file1 (DOCX 37 kb)

## Data Availability

Data generated and analysed during this study are available from the corresponding author upon reasonable request.

## Material availability

Data generated and analysed during this study are available from the corresponding author upon reasonable request.
