# Supplementary material for: Effects on clients' daily functioning and common features of reablement interventions: a systematic literature review
Source: Eur J Ageing. 2022 May 3;19(4):903–29. doi: 10.1007/s10433-022-00693-3 (PMC9729664; doi:10.1007/s10433-022-00693-3)
Supplement: Supplementary file 1 — Supplementary file1 (DOCX 37 kb) [file 10433_2022_693_MOESM1_ESM.docx]

# Appendix 1 - Search strategy

**PubMed**

Date: July 24 2020

Limits: 2002–2020; English and Dutch

Update: July 23, 2021

#1 Aged [mh] OR Aging [mh] OR geriatrics [mh] OR Frail elderly [mh] OR geriatric* [tiab] OR elder* [tiab] OR old [tiab] OR older [tiab] or ageing [tiab] or aging [tiab]

#2 Home care services [mh] OR home care service* [mh] OR rehabilitation [tiab] OR enablement [tiab] OR reablement [tiab] OR function focused care* [tiab] OR restorative care* [tiab]

#3 Activities of daily living [mh] OR independent living [mh] OR self care [mh] OR recovery of function [mh] OR self care [tiab] OR recovery of function* [tiab] OR disab*[tiab] OR daily function* [tiab] OR activities of daily living [tiab] OR instrumental activities of daily living [tiab] OR iADL [tiab] OR ADL [tiab] OR autonomy* [tiab] OR physical function* [tiab] OR functional status [tiab]) OR functional improvement [tiab]) OR functional decline [tiab] OR improving abilit* [tiab] OR independent living [tiab] OR independen* [tiab] OR "dependen* [tiab]

#4 randomized controlled trial [Publication Type] OR controlled clinical trial [Publication Type] OR randomized [tiab] OR placebo [tiab] OR Clinical Trials as Topic [mh] OR randomly [tiab] OR trial [Title]

#5 animals [mh] NOT humans [mh]

#6 #1 AND #2 And #3 and #4 NOT #5

**EBSCO (CINAHL)**

Date: July 24, 2020

Limits: 2002–2020; English and Dutch

Update: July 23, 2021

#1 (MH Aging) OR (MH Aged) OR (MH Geriatrics) OR (MH Frail elderly) OR TI (aged OR geriatric* OR elder* OR old OR older OR ageing OR aging) OR AB (aged OR geriatric* OR elder* OR old OR older OR ageing OR aging)

#2 TI (home care service* OR rehabilitation* OR enablement OR reablement* OR function focused care* OR restorative care*) OR AB (home care service* OR rehabilitation* OR enablement OR reablement* OR function focused care* OR restorative care*)

#3 (MH Activities of Daily Living) OR (MH self care) OR TI ((independent living) OR (recovery of function) OR (self care) OR disab* OR (daily function*) OR (activities of daily living) OR (instrumental activities of daily living) OR iADL OR ADL OR autonom* OR (physical function*) OR (functional status) OR (functional improvement) OR (functional decline) OR (improving abilit*) OR independen* OR dependen*) OR AB ((independent living) OR (recovery of function) OR (self care) OR disab* OR (daily function*) OR (activities of daily living) OR (instrumental activities of daily living) OR iADL OR ADL OR autonom* OR (physical function*) OR (functional status) OR (functional improvement) OR (functional decline) OR (improving abilit*) OR independen* OR dependen*)

#4 (MH randomized controlled trials) OR (MH double-blind studies) OR (MH single-blind studies) OR (MH random assignment) OR (MH pretest-posttest design) OR (MH cluster sample) OR TI (randomised OR randomized) OR AB (random*) OR TI (trial) OR (MH (sample size) AND AB (assigned OR allocated OR control)) OR MH (placebos) OR PT (randomized controlled trial) OR AB (control W5 group) OR (MH (crossover design) OR MH (comparative studies)) OR AB (cluster W3 RCT)

#5 (((MH animals+ OR MH (animal studies) OR TI (animal model*)) NOT MH (human))

#6 #1 AND #2 AND #3 AND #4 NOT #5

**EBSCO (Psycinfo)**

Date: July 24, 2020

Limits: 2002–2020

Update: July 23, 2021

#1 (DE Aging) OR (DE Aged) OR (DE Geriatrics) OR (DE Frail elderly) OR TI (aged OR geriatric* OR elder* OR old OR older OR ageing OR aging) OR AB (aged OR geriatric* OR elder* OR old OR older OR ageing OR aging)

#2 TI (home care service* OR rehabilitation* OR enablement OR reablement* OR function focused care* OR restorative care*) OR AB (home care service* OR rehabilitation* OR enablement OR reablement* OR function focused care* OR restorative care*)

#3 (DE Activities of Daily Living) OR (DE self care) OR TI ((independent living) OR (recovery of function) OR (self care) OR disab* OR (daily function*) OR (activities of daily living) OR (instrumental activities of daily living) OR iADL OR ADL OR autonom* OR (physical function*) OR (functional status) OR (functional improvement) OR (functional decline) OR (improving abilit*) OR independen* OR dependen*) OR AB ((independent living) OR (recovery of function) OR (self care) OR disab* OR (daily function*) OR (activities of daily living) OR (instrumental activities of daily living) OR iADL OR ADL OR autonom* OR (physical function*) OR (functional status) OR (functional improvement) OR (functional decline) OR (improving abilit*) OR independen* OR dependen*)

#4 TI (double-blind OR (random* assigned) OR control) OR AB (double-blind OR (random* assigned) OR control)

#5 #1 AND #2 AND #3 AND #4

**Cochrane**

Date: July 24, 2020

Limits: 2002–2020

Update: July 23, 2021

#1 ((aged OR geriatric* OR elder* OR old* OR ageing OR aging OR frail elderly).ti,ab) (Word variations have been searched)

#2 MeSH descriptor: [Frail Elderly] explode all trees

#3 MeSH descriptor: [Aging] explode all trees

#4 MeSH descriptor: [Geriatrics] explode all trees

#5 MeSH descriptor: [Aged] explode all trees

#6 #1 OR #2 OR #3 OR #4 OR #5

#7 ((home care service* OR rehabilitation OR enablement OR reablement* OR function focused care OR restorative care).ti,ab) (Word variations have been searched)

#8 MeSH descriptor: [Home Care Services] explode all trees

#9 #7 OR #8

#10 ((self care OR recovery of function* OR disab* OR daily function* OR activities of daily living OR instrumental activities of daily living OR iADL OR ADL OR autonom* OR physical function* OR functional status OR functional improvement OR functional decline OR improving abilit* OR independent living OR independen* OR dependen*).ti,ab) (Word variations have been searched)

#11 MeSH descriptor: [Activities of Daily Living] explode all trees

#12 MeSH descriptor: [Independent Living] explode all trees

#13 MeSH descriptor: [Self Care] explode all trees

#14 MeSH descriptor: [Recovery of Function] explode all trees

#15 #10 OR #11 OR #12 OR #13 OR #14

#16 #6 AND #9 AND #15

# Appendix 2 – Data extraction template

**Study characteristics**

| *Author* | *Year* | *Country* | *Quality* | *Title* | *Study aim* | *Hypotheses* | *Study design* | *Setting* | *Study sample* | *Baseline characteristics* | *Outcome measures* | *Limitations* | *Study protocol / related publications* |
| --- | --- | --- | --- | --- | --- | --- | --- | --- | --- | --- | --- | --- | --- |
|  |  |  |  |  |  |  |  |  |  |  |  |  |  |

**Intervention content**

| *Author* | *Year* | *Aims* | *Characteristics*  *(intensive, person-centred, interdisciplinary, coordinated)* | *Components*  *(assessment, goal-oriented, regular reassessment, training of daily activities, use of home modifications/assistive devices, involvement social network, reablement training and support of staff)* | *Target group (diagnosis, age, physical capacity, setting, type of problem)* | *Results* |
| --- | --- | --- | --- | --- | --- | --- |
|  |  |  |  |  |  |  |

**Outcomes**

| *Study* | *Outcome measures* | *Results Follow-up, Mean (95% CI), p* |  |
| --- | --- | --- | --- |
|  |  |  |  |
